# Postoperative extracranial metastasis from glioblastoma: a case report and review of the literature

**DOI:** 10.1186/s12957-017-1300-7

**Published:** 2017-12-29

**Authors:** Wenjiao Wu, Dequan Zhong, Zhan Zhao, Wentao Wang, Jun Li, Wei Zhang

**Affiliations:** 1 Department of Neurosurgery, The First Affiliated Hospital of Jinan University, Guangzhou, China; 2 Neurosurgical Research Institute, The First Affiliated Hospital of Guangdong Pharmaceutics University, Guangzhou, 510060 China; 3 Guangdong Province Key Laboratory of Brain Function and Disease, Department of Biochemistry, Zhongshan School of Medicine, Sun Yat-sen University, Guangzhou, China

**Keywords:** Glioblastoma, Extracranial metastasis, Case report

## Abstract

**Background:**

Glioblastoma is the most common primary malignant brain tumor. Extraneural metastases are rarely reported in the literature.

**Case presentation:**

We report a case of a 38-year-old patient who was diagnosed with glioblastoma in 2015. Four months after surgery, local relapse was found and the patient received a second surgery. After another 4 months, we found a hard mass in the right posterior neck when she admitted to our department for fourth cycle of adjuvant chemotherapy. Immunohistochemical investigation supported the diagnosis of glioblastoma metastases to the neck after resection of the right neck mass. A few days later, spinal vertebral magnetic resonance imaging (MRI) confirmed multiple metastases in the thoracic, lumbar, sacral, and bilateral iliac bones.

**Conclusions:**

Glioblastoma is the most common primary malignant brain tumor. Whole tumor resection and early radiotherapy and chemotherapy can delay recurrence and prolong survival. Extracranial metastases are extremely rare. We report this case with the aim of bringing attention to extracranial metastasis of brain glioma.

## Background

Glioblastoma is the most common primary malignant tumor in the neural tissue, with an incidence of 3.19/100,000 patient-years [1]. The tumor is defined as grade IV according to the World Health Organization (WHO) classification [2]. The treatment of choice currently consists of maximal safe surgical resection and postoperative concomitant radiochemotherapy, followed by chemotherapy with the orally administered alkylating drug temozolomide [3]. The median survival time is 12 months [2]. Historically, glioblastomas were not believed to metastasize outside of the central nervous system (CNS) because of the blood–brain barrier and overall low median survival. However, in 1928, Davis reported a patient with disseminated glioblastoma [4]. Several reports of extraneural glioblastoma metastases have since been published. In 2015, Pietschmann et al. reviewed existing data and performed a new meta-analysis of 150 cases [5]. The incidence of extraneural metastases among all patients with glioblastoma is 0.2% [6].

## Case presentation

A 38-year-old woman presented paroxysmal headache for 1 month. She underwent brain computed tomography (CT) scan, which showed a space-occupying lesion in the right temporal lobe. The patient underwent surgery; the histological diagnosis was glioblastoma (WHO grade IV). Chemotherapy and radiotherapy were not administered after surgical operation. No other examinations were performed for 3 months, during which she gradually developed dizziness and headaches. Brain MRI revealed local recurrence, with multiple tumors in the right temporal–occipital junction. A second surgery was performed successfully; the histological diagnosis was also glioblastoma (WHO grade IV). The patient received adjuvant radiotherapy (64 Gy in 6 weeks using LINAC) and adjuvant chemotherapy with temozolomide, first concomitantly to radiotherapy (75 mg/m^2^/day), and then sequentially (200 mg/m^2^/day for 5 days per cycle of 28 days, for 3 cycles).

**Fig. 1:**
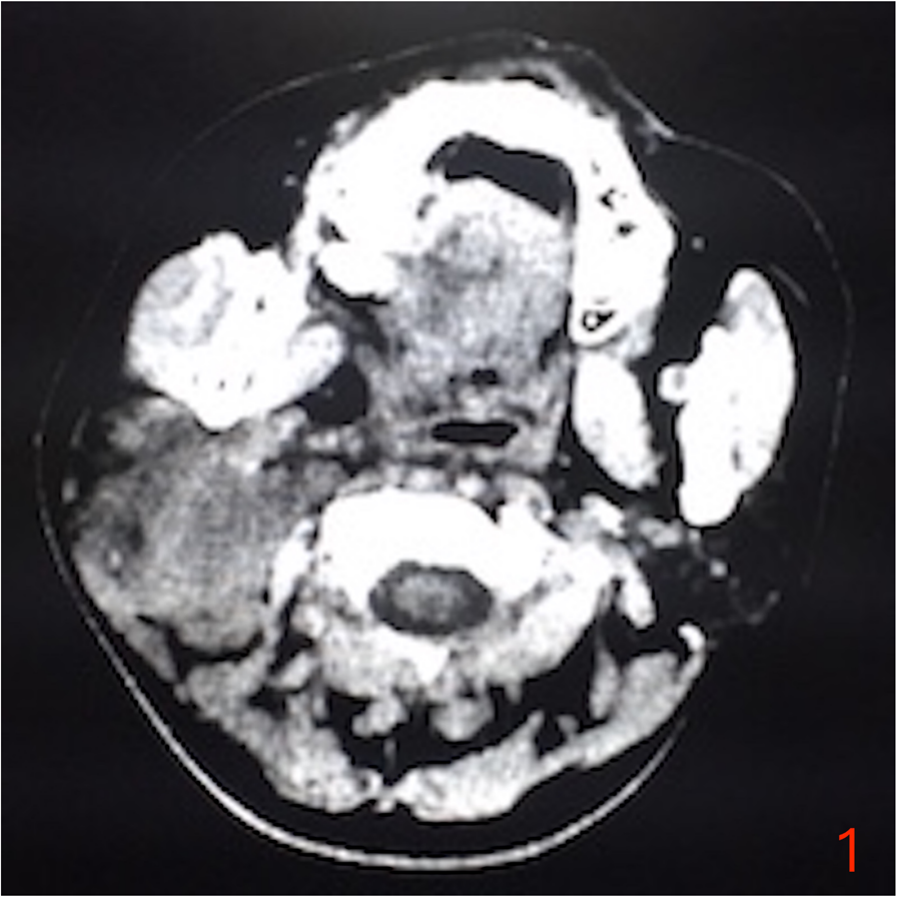
Enhanced neck CT showing multiple swollen lymph nodes of varying sizes fused into a 54 × 39 × 83 mm mass with irregular edges and heterogeneous enhancement

**Fig. 2:**
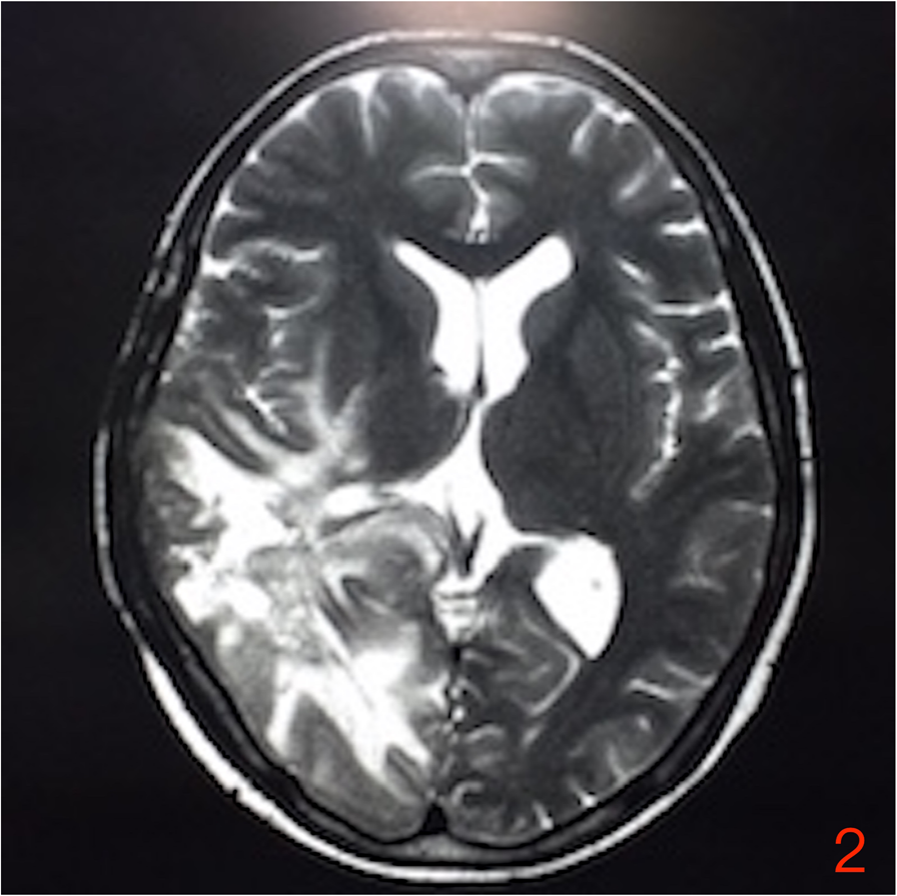
Head MRI showing multiple occupations in the right temporal, apical, and occipital lobes and in the corpus callosum

**Fig. 3:**
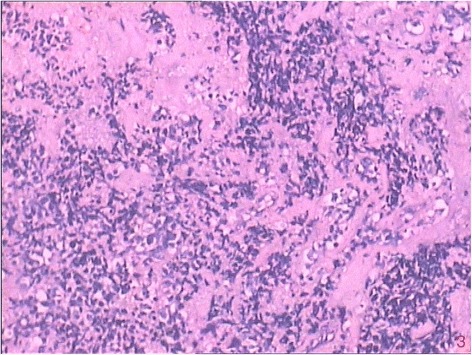
Postoperative immunohistochemical investigation supporting the diagnosis of glioblastoma metastases to the neck, WHO grade IV. (H&E, ×400)

**Fig. 4:**
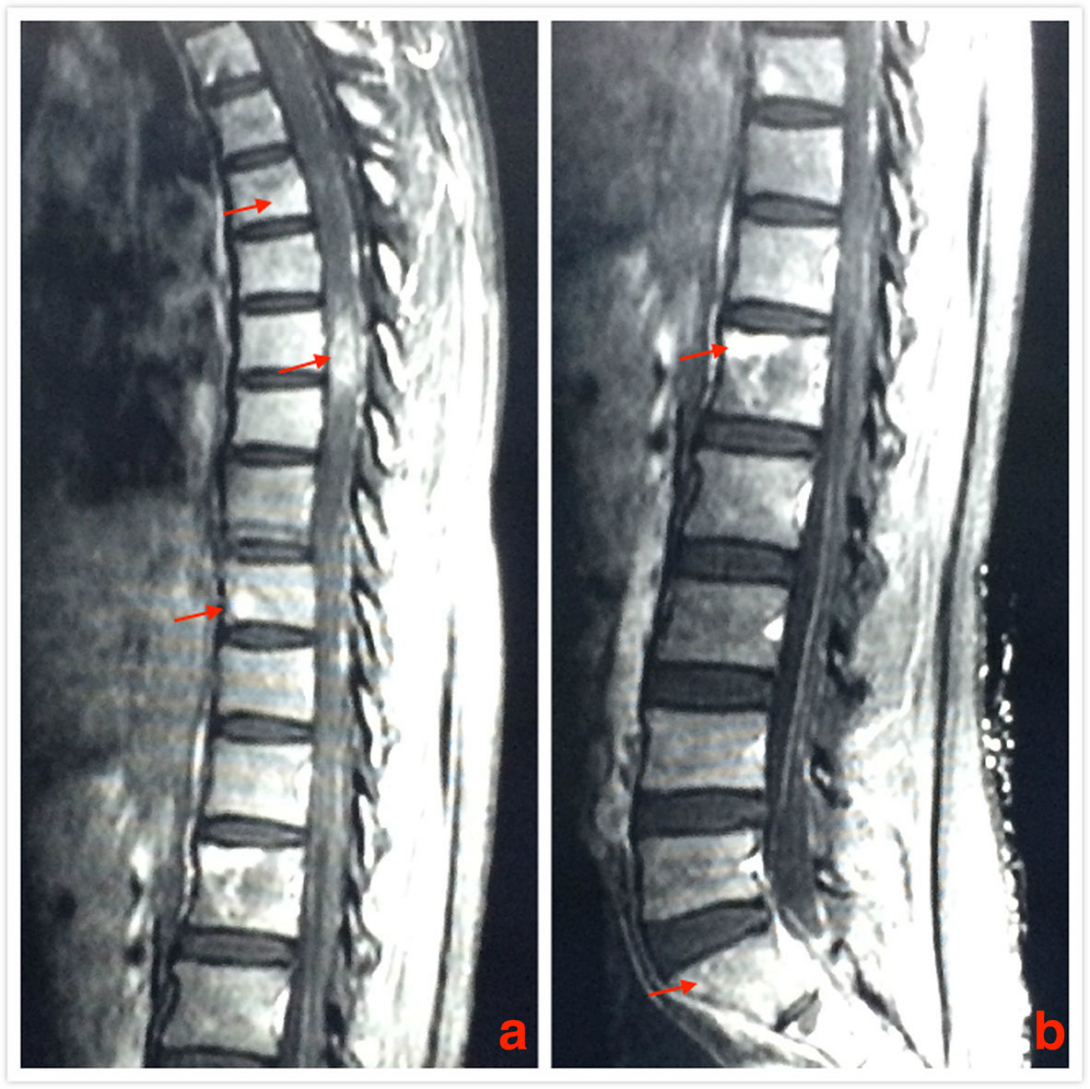
Spinal vertebral MRI confirming multiple metastases in the thoracic (**a**) and lumbar (**b**)

On July 16, 2016, the patient, suffering from head and neck pain for 2 days, was admitted to our department for fourth cycle of adjuvant chemotherapy. Physical examination revealed a hard mass with unclear boundaries in the right posterior neck. Neck CT revealed multiple swollen lymph nodes of varying sizes fused into a 54 × 39 × 83 mm mass with irregular edges and heterogeneous enhancement by enhanced scan, which was deemed metastases (Fig. 1). Head MRI revealed multiple occupations in the right temporal, apical, and occipital lobes and in the corpus callosum consistent with tumor postoperative recurrence (Fig. 2). To clarify the nature of the lumps, puncture biopsy was performed on day 6 after admission. Histological and immunohistochemical investigation revealed malignant tumor with the characteristics of glioma. As nerve compression by the mass caused severe neck pain in the patient, we planned surgical treatment to alleviate the pain symptoms and to further clarify the pathology. On July 30, 2016, the patient underwent resection of the right neck mass. Surgery revealed that the mass was deep in the front of the sternocleidomastoid muscle, with a hard texture and close adhesion to the surrounding tissue. Postoperative immunohistochemical investigation supported the diagnosis of glioblastoma metastases to the neck, WHO grade IV (Fig. 3). The patient’s neck pain was significantly alleviated after surgery, but 4 days after surgery, she developed pain in the waist and right lower limbs. Spinal vertebral MRI confirmed multiple metastases in the thoracic, lumbar, sacral, and bilateral iliac bones (Fig. 4a and b). As the waist and right lower limb pain gradually increased, symptomatic treatment became invalid. In light of the rapid progress of the tumor, we took more drastic treatment measures, but there was no obvious improvement. On August 12, 2016, the patient’s relatives requested that she be discharged back to the local primary hospital for conservative treatment. Two occasions of follow-up by telephone on August 22, 2016, and September 10, 2016, revealed further deterioration of the patient’s condition, and she died of systemic organ failure on September 6, 2016.

## Discussion

Glioblastoma is the most common primary malignant brain tumor, with the characteristic of a high degree of anaplastic growth. The average survival time is about 9–10 months. Glioblastoma also has the characteristics of rapid postoperative recurrence and poor prognosis, rendering it a serious threat to human health. Currently, it is considered one of the most difficult tumors to treat in neurosurgical management.

Glioblastoma extracranial metastasis is extremely uncommon; the main reasons for which may include (1) strong protective mechanisms in the CNS, such as the lack of a true lymphatic system in the brain and the venous sinuses encased in dense dural membranes hindering invasion [7, 8], and (2) patients with glioblastoma, who have a short survival time, may die from oncothlipsis, intracranial hypertension, or other complications before extracranial metastasis develops [9]. However, various types of glioma extracranial metastasis have been reported since 1928, when Davis first reported glioma meningeal metastasis [4]. The most common metastasis sites are the lymph nodes and the lungs, spinal cord, and bone [10]. Glioblastoma is more common than other primary intracranial tumors with extracranial metastasis. Liwnicz and Rubinstein [11] analyzed 116 cases in the literature and found that the commonest metastasizing glioma tumor type was glioblastoma (41.4%), followed by medulloblastoma (26.7%), ependymoma (16.4%), astrocytoma (10.3%), and oligodendroglioma (5.25%).

In clinical work, the diagnosis must be strictly defined if the report is to be considered an acceptable case of metastasizing CNS glioma. Weiss [12] proposed the following diagnostic criteria in 1955: (1) the presence of a single histologically characteristic tumor of the CNS must be proven. (2) The clinical history must indicate that initial symptoms were due to this tumor. (3) A complete autopsy must be performed and reported in sufficient detail to rule out the possibility of any other primary tumor site. (4) The morphology of the CNS tumor and of the distant metastases must be identical with due allowance for differences in degree of anaplasia. Although the combination of noninvasive scanning techniques, stereotactic biopsy, and immunohistochemical techniques has rendered autopsy no longer necessary, the diagnostic criteria still have certain value. Four months after the second operation and radiotherapy and chemotherapy, our patient developed a hard tissue mass in the right side of the neck. The postoperative pathological morphology and histology of the mass were consistent with that of the primary intracranial tumor and were also consistent with the diagnosis of cervical glioblastoma lymph node metastasis. At the same time, further examination revealed that the patient had metastatic lesions in the thoracic, lumbar, sacral, and bilateral iliac vertebrae; spinal cord; and bone marrow. Although no relevant pathological examination was performed, her medical history and physical and imageological examination results led us to consider these metastases from the brain glioblastoma.

To date, the exact mechanism of extracranial metastasis of glioblastoma remains unclear, but the extracranial metastasis may be related to the following factors: (1) age at the first diagnosis: Piccirilli et al. [13] reviewed the literature from 1928 to 2006 and reported on 128 cases of extracranial metastases of glioblastoma in 2008. The average age of the patients at presentation was 40 years; however, the mean age at the first diagnosis of other patients with glioblastoma was 54 years [14]. From this, we can conclude that young glioblastoma patients have the potential to develop extraneural metastasis. (2) Longer lifespan: with a higher level of treatment, a higher index of suspicion, and better diagnostic tools, patients with glioblastoma now have longer survival times, which in turn lead to the potential for developing extraneural metastases. At the same time, the meta-analysis support the idea that prolonged survival of patients increases the probability of glioblastoma cells shedding to lymphatic and hematic systems [15]. (3) Surgical intervention: nearly all (96%) reported patients with extraneural metastases had undergone prior cranial surgery [16]. A requirement of extracranial metastasis is tumor cells crossing the dura mater, which is the most important barrier. Surgery may lead to dural damage and potentially facilitate extracranial extension [17]. So the operation should strictly adhere to the tumor-free principle and try to guarantee completeness of cerebral dura mater for the sake of reducing the occurrence rate of extracranial metastasis [18]. Metastases also occur in the abdominal cavity after ventriculoperitoneal shunt [19], and glioma cells can spread along the biopsy pathways [20]. (4) Spontaneous tumor metastasis: although surgery is an important condition for the occurrence of metastasis, prior surgery was absent in some cases of extracranial metastases [21]. This may be related to tumor chemoradiotherapy. Tuettenberg et al. [22] recently proposed and proved the mechanism of glioma angiogenesis inhibition and escape in radiotherapy and chemotherapy, where tumor angiogenesis was inhibited and primary tumor growth was inhibited, but tumor cell invasion and proliferation adjacent to the brain tissue was increased.

There is still a lack of effective treatment for extracranial metastatic brain glioma, and the prognosis is very poor [21]. Therefore, whether total tumor resection is performed would not have an obvious effect on postoperative survival, but the degree of tumor resection is an important influencing factor of postoperative radiotherapy and chemotherapy [23]. More residual tumor results in a more obvious decline in the effect of radiotherapy and chemotherapy. For high-grade gliomas, removing as much tumor tissue as possible surgically and early radiotherapy and chemotherapy can play a role in killing the residual tumor cells, delaying the tumor recurrence time and prolonging survival. On the other hand, there should be increased awareness among surgeons for preventing tumor implantation or metastasis and attempting to reduce damage to the surrounding tissue. Once a space-occupying lesion has been identified, the potential for tumor metastasis should be considered. Thoracic abdominal CT and lymph node and bone scans are necessary, as the most common metastasis sites are the lymph nodes, spinal cord, bone, and liver. Pathological examination could also have a greatly significant effect on clear diagnosis. When metastatic lesions are found, timely and correct treatment should be administered to avoid increased metastases and even further spread to other systems.

## Conclusions

Glioblastoma is the most common primary malignant brain tumor. Although extracranial metastases remain extremely uncommon, extraneural spread of the tumor is possible and must be considered. Although we have described extracranial metastases of malignant gliomas in detail, the complex mechanism of metastasis remains unclear. Whole tumor resection and early radiotherapy and chemotherapy can delay recurrence and prolong survival. We report this case with the aim of bringing attention to extracranial metastasis of brain glioma.

## Abbreviations

CNS: Central nervous system
CT: Computed tomography
LINAC: Linear accelerator
MRI: Magnetic resonance imaging
WHO: World Health Organization
